# Supplementary figures and images for: Hematological and cytochemical characteristics of peripheral blood cells in the argus snakehead (Ophiocephalus argus Cantor)
Source: PeerJ. 2021 Apr 26;9:e11234. doi: 10.7717/peerj.11234 (PMC8083180; doi:10.7717/peerj.11234)

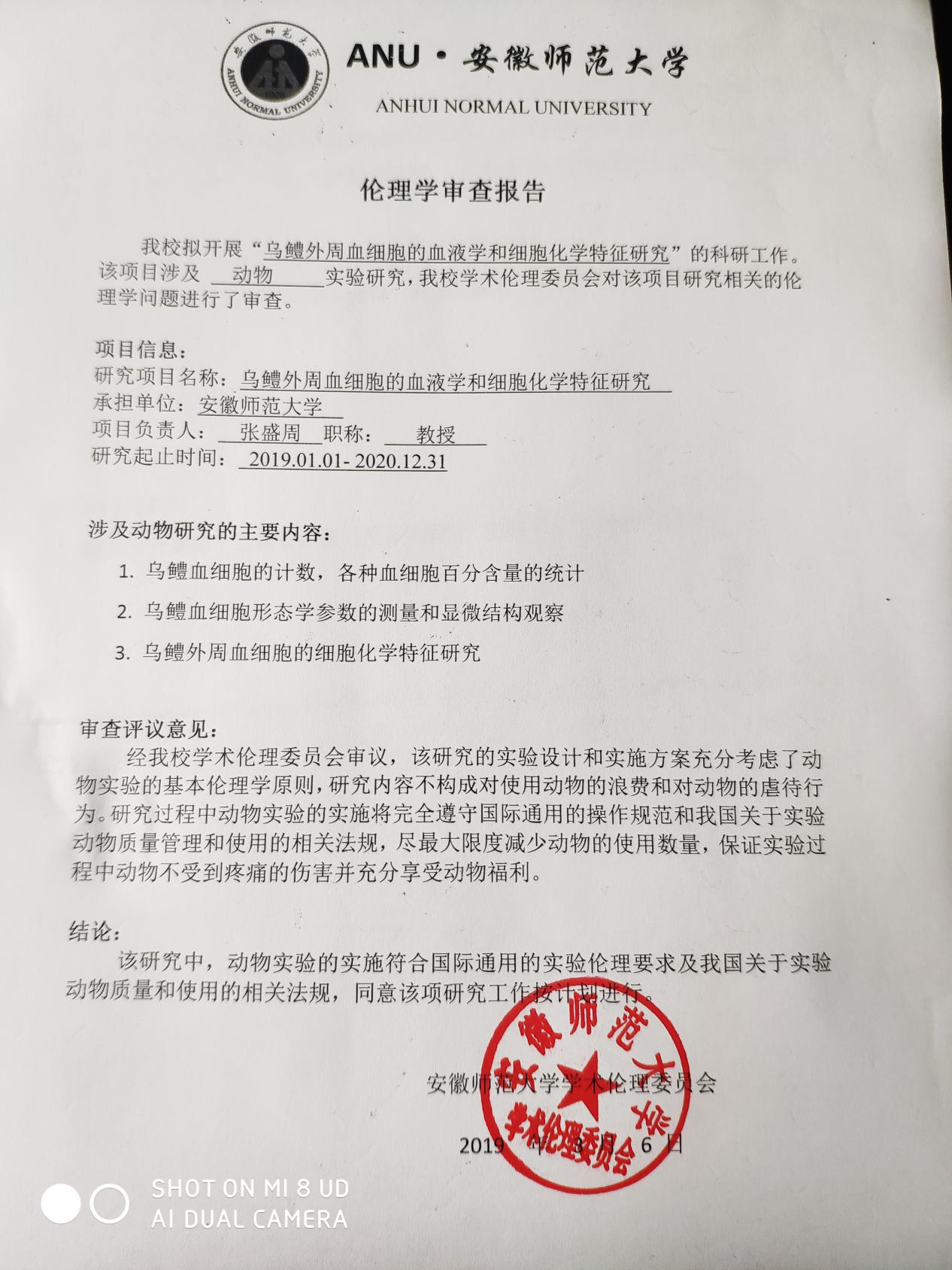

Supplement: File S6 — Original cytochemical staining images. [file peerj-09-11234-s006.docx]
